# An FMRFamide Neuropeptide in Cuttlefish *Sepia pharaonis*: Identification, Characterization, and Potential Function

**DOI:** 10.3390/molecules25071636

**Published:** 2020-04-02

**Authors:** Yang Zhu, Lian-lian Sun, Jun-hong Wu, Hui-hui Liu, Li-bing Zheng, Zhen-ming Lü, Chang-feng Chi

**Affiliations:** National and Provincial Joint Laboratory of Exploration and Utilization of Marine Aquatic Genetic Resources, National Engineering Research Center of Marine Facilities Aquaculture, School of Marine Science and Technology, Zhejiang Ocean University, 1st Haidanan Road, Changzhi Island, Lincheng, Zhoushan 316022, China

**Keywords:** neuropeptide, FMRFamide, *Sepia pharaonis*, cuttlefish

## Abstract

Neuropeptides are released by neurons that are involved in a wide range of brain functions, such as food intake, metabolism, reproduction, and learning and memory. A full-length cDNA sequence of an *FMRFamide* gene isolated from the cuttlefish *Sepia pharaonis* (designated as *SpFMRFamide*) was cloned. The predicted precursor protein contains one putative signal peptide and four FMRFamide-related peptides. Multiple amino acid and nucleotide sequence alignments showed that it shares 97% similarity with the precursor FMRFamides of *Sepiella japonica* and *Sepia officinalis* and shares 93% and 92% similarity with the *SpFMRFamide* gene of the two cuttlefish species, respectively. Moreover, the phylogenetic analysis also suggested that *Sp*FMRFamide and FMRFamides from *S. japonica* and *S. officinalis* belong to the same sub-branch. Tissue expression analysis confirmed that *SpFMRFamide* was widely distributed among tissues and predominantly expressed in the brain at the three development stages. The combined effects of *Sp*FMRFamide+*Sp*GnRH and *Sp*FLRFamide+*Sp*GnRH showed a marked decrease in the level of the total proteins released in the CHO-K1 cells. This is the first report of *SpFMRFamide* in *S. pharaonis* and the results may contribute to future studies of neuropeptide evolution or may prove useful for the development of aquaculture methods for this cuttlefish species.

## 1. Introduction

*Sepia pharaonis* is an economically significant cephalopod that plays an important role in the marine ecosystem. It is famous for its delicacy and value meat; the species is also noted for its potential medicinal value [1]. However, *S. pharaonis* resources have declined since the 1980s due to overfishing, environmental damage, and habitat degradation. *S. pharaonis* is an endangered species [2]; because of this situation, our research group began to work on resource restoration. We found that the sexual maturity of artificially cultured cuttlefish starts earlier than that of the wild ones, and due to this phenomenon, the cultured cuttlefish is susceptible to precocious puberty [2]. The peptide under consideration here is known to be involved in reproduction and thus is closely related to the sexual maturity of these cephalopods. The recovery and utilization of cuttlefish resources rely on the continued studies of the reproductive characteristics of the species. Neuropeptides, widely distributed in both invertebrates and vertebrates, usually act as neurotransmitters, neuromodulators, and neurohormones. FMRFamide-like peptides (FLPs), the largest and most diverse family of known neuropeptides [3], represented by the tetrapeptide FMRFamide (Phe-Met-Arg-Phe-NH2; FMRFa), share high sequence similarity with FMRFamide and are sometimes also referred to as FMRFamide-related peptides (FaRPs).

FLPs are defined as peptides that end in Arg-Phe-NH2 [3]. FaRPs are defined as those that are evolutionarily related to FMRFa. However, it is difficult to elucidate the true evolutionary homology of FLPs and FMRFa, because diverse ranges of peptides across the animal kingdom share the C-terminal RFamide motif [4,5]. Following the identification of the founder FMRFamide sequence from the clam *Macrocallista nimbosa* [6], a number of researches on FLPs were carried out in metazoans [7]. In metazoans, different FLP types may exist in the same species, and the same FLP type may occur in various species [5]. FLPs could intimately take part in a broad pattern of biological processes, including feeding, cardiovascular function, and water homeostasis [4,5,8,9]. For instance, FMRFamide, FLRFamide, and two types of generalized FLPs (GDPFLRFa and GGKYMRFa) are present in annelids [10,11]. In cnidarians, GRFa peptides that are secreted by nerve cells contained within a nerve net [12] are likely to participate in metamorphosis, food capture, locomotion, and defense [13].

Nowadays, only generalized FLPs (GDPFLRFa and GGKYMRFa) have been identified in arthropods [14] and in insects [15,16], however, FLP types which are found in crustaceans with amino acid residues at N-terminals may be FLRFamides [5]. FMRFamide-like immunoreactivity has been detected in the central neurons of leeches (*Hirudo medicinalis*) [17], and physiological experiments showed that FLPs could regulate the cardiac function of leeches [18]. Reports of FLPs in nematodes (*Caenorhabditis elegans*) have identified more than 70 types of FLPs from approximately 33 genes [19,20], and those FLPs might play vital roles in reproduction [21], feeding, and clustering activity [22]. Furthermore, Sa-112, which is highly homologous to SchistoFLRFamide [23], inhibited contraction of the locust oviduct, which is a typical activity of FMRFamide peptides.

At present, more than 40 types of FLPs from the five major mollusk classes (Cephalopoda, Scaphopoda, Bivalvia, Gastropoda, and Polyplacophora) have been isolated and characterized [5,24]. Furthermore, several physiological assays have demonstrated that these FLPs might be involved in food intake [25,26], reproduction [26,27], and heart activity [28]. Moreover, an FMRFamide expressed by a single gene in cephalopods was identified as a member of the FaRP subfamily [28,29]. Regarding vertebrates, just one peptide with FMRFamide or FLRFamide present at the C-terminal has been found in frogs [30], which suggests that the mechanisms of FLPs in vertebrates regulating the expression or the physiological systems are more complex than those of invertebrates. However, FLPs found in vertebrates also possess several physiological functions such as control of insulin secretion [31], daily food intake [32], regulation of the reproductive axis [33], and precipitation of opiate-withdrawal syndrome [34]. These data provided the evidence that the vertebrate cells were responding to these neuropeptides.

This study represents the first report describing the full-length sequence of an FMRFamide precursor in *S. pharaonis* (hereafter referred to as *SpFMRFamide*). Moreover, quantitative real-time PCR (qRT-PCR) and in vitro assay with CHO-K1 cells were used to assess the possible function of *Sp*FMRFamide in *S. pharaonis*. These results will clarify the function and possible role of FMRFamide in the development of *S. pharaonis* individuals.

## 2. Results

### 2.1. Full-Length Transcript Sequence and Predicted Precursor Protein

**Figure 1:**
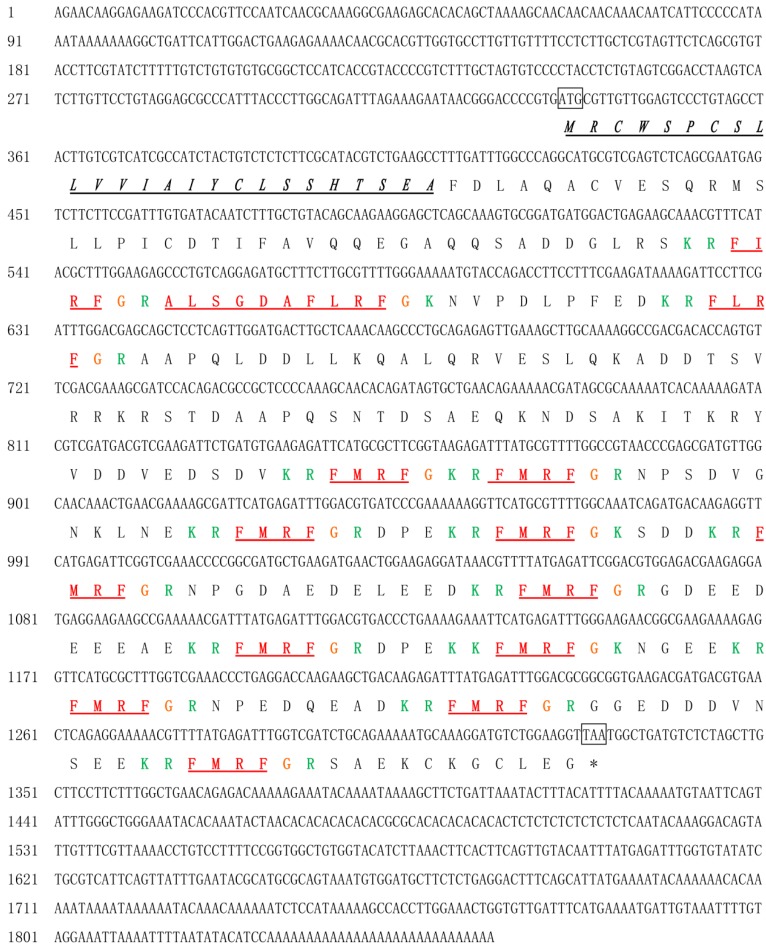
Nucleotide sequence and deduced amino acid sequence of *Sp*FMRFamide precursor. The initiation codon (ATG) and stop codon (TAA) are marked. The putative signal peptide is underlined in black and italic, and the predicted RFaRPs are marked in red. The basic cleavage sites are identified green color, and the glycines used for C-terminus amidation are marked in orange.

We isolated, cloned, and sequenced the cDNA from *S. pharaonis*, and the gene was named *SpFMRFamide*. The whole sequence of *SpFMRFamide* cDNA was 1856 bp (GenBank accession No. KX000397), with a 996 bp open reading frame (ORF) encoding protein precursor of 331 amino acid (aa) (Figure 1). The start codon ATG was located at nucleotides 335–337, and the stop codon TAA was located at 1328–1330. There were 334 base pairs of untranslated region (UTR) in the 5′ end as well as 526 base pairs of UTR in the 3′ end. The predicted precursor protein contained a putative signal peptide of 24 aa and four distinct FMRFamide-related peptides: thirteen tetrapeptide (one copy of FIRFamide, one copy of FLRFamide, and eleven copies of FMRFamide), and one decapeptide (ALSGDAFLRFamide). FaRPs ending with RF residues and being amidated post-translationally at the C-terminus, with a glycine immediately behind the RF motif, may be considered to be related to the post-translational amidation of arginine-phenylalanine. Lysine-arginine/lysine residues at the N-terminus and lysine-arginine or arginine/lysine at the C-terminus might serve as internal proteolytic cleavage sites for each FaRP during post-translational processing. The predicted entire precursor protein theoretical molecular weight (MW) was 35.91 kDa lacking signal peptide, and the estimated *pI* was 9.09. Blastn analysis showed that *Sp*FMRFamide shared a 97% sequence similarity with *Sj*FMRFamide (KJ933411) and a precursor FMRFamide from *Sepia officinalis* (P91889.1).

### 2.2. Blast Search and Phylogenetic Tree Construction

**Figure 2:**
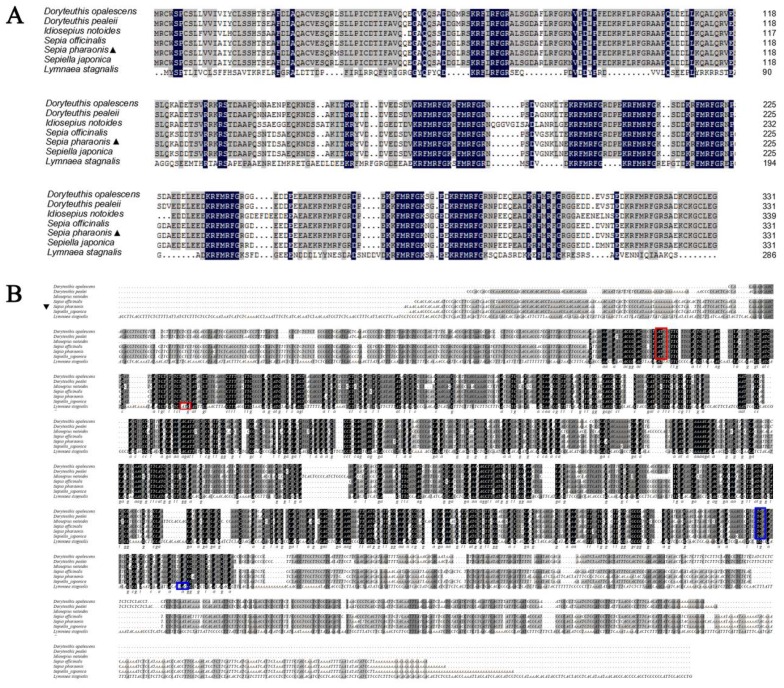
Multiple amino acid (**A**) and nucleotide (**B**) sequence alignment of the FMRFamide precursor of *Doryteuthis opalescens* (Q9GSL0.1, AF303160.1), *D. pealeii* (B6E465.1, FJ205479.1), *Idiosepius notoides* (ACP39631.1, FJ896403.1), *Sepia officinalis* (P91889.1, Y11246.1), *Sepiella japonica* (KJ933411, KJ933411.1), *Lymnaea stagnalis* (AAA63280.1, M87479.2), in comparison with *S. pharaonis* which was noted by ▲. Residues identical in all seven sequences are boxed in black. Conservative substitutions are boxed in gray. The red box represents the initiation codon and the blue box represents the termination codon.

To reveal homologous amino acid sequences, the predicted *Sp*FMRFamide precursor protein was aligned with those of the other species. The results indicated that the *Sp*FMRFamide protein shared 97%, 97%, 92%, 92%, 84%, and 41% identity with the FMRFamide precursors of *Sepiella japonica*, *S. officinalis*, *Doryteuthis pealeii*, *D. opalescens*, *Idiosepius notoides*, and *Lymnaea stagnalis*, respectively (Figure 2A). Thus, it is seen that the primary structure of the *Sp*FMRFamide precursor is highly conserved. Meanwhile, to reveal homologous nucleotide sequences and show any putative cis-acting elements involved in the expression of the precursor protein, the full nucleotide sequence of the *SpFMRFamide* gene was aligned with those of the other species, and the results indicated that the *SpFMRFamide* gene shared 93%, 92%, 83%, 77%, 72%, and 43% identity with the *FMRFamide* genes of *S. japonica*, *S. officinalis*, *D. pealeii*, *L. stagnalis*, *D. opalescens*, and *I. notoides*, respectively (Figure 2B). Thus, it is seen that the sequence conservation of the *Sp*FMRFamide gene is relatively highly conserved and the similarity of the nucleotide sequences was lower than the amino acid one.

**Figure 3:**
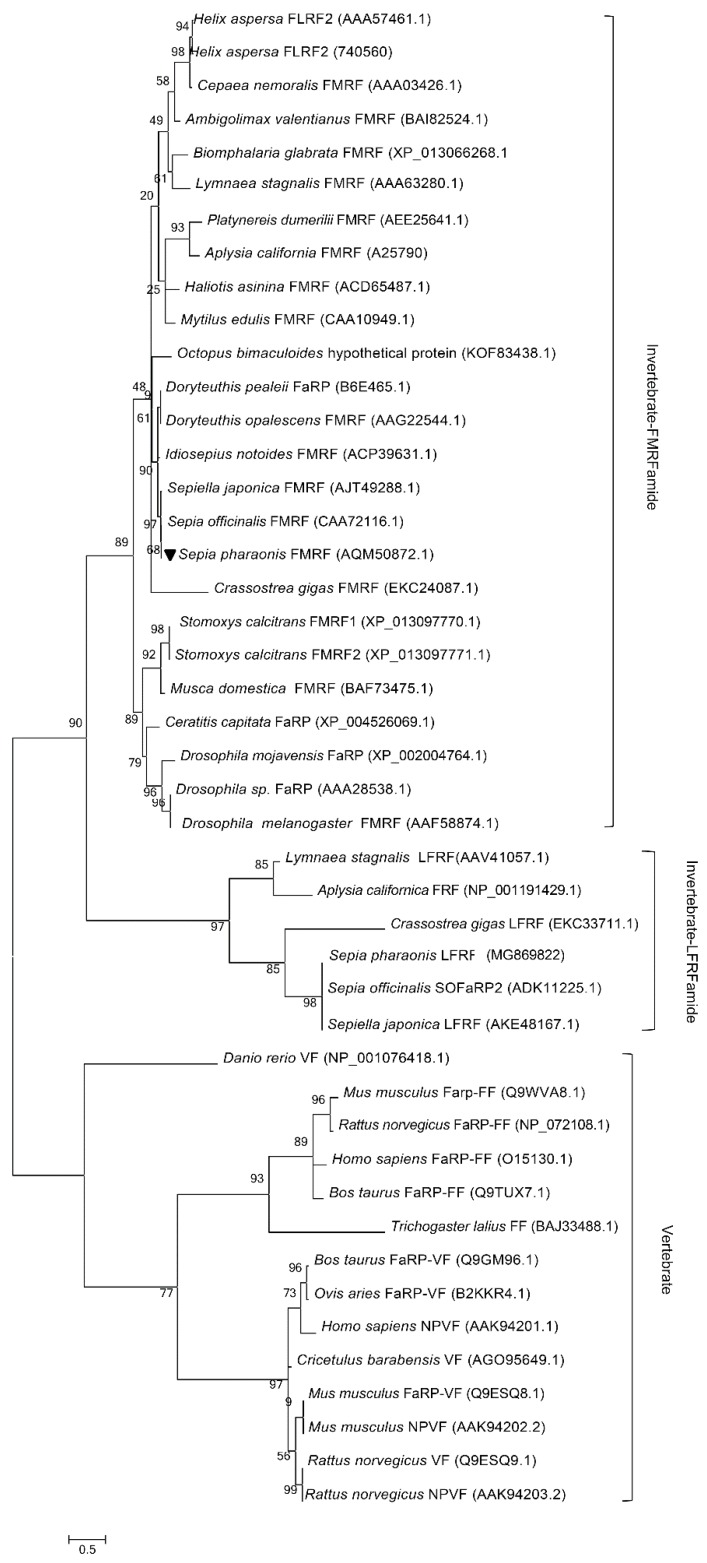
Phylogenetic tree constructed with *Sp*FMRFamide which was noted by ▼ and 44 kinds of other identified RFamide precursors by the maximum likelihood method (MEGA 6.0). All protein sequences were obtained from GenBank of the US National Center for Biotechnology Information (NCBI), and the GenBank accession numbers are in parentheses. The topological stability of the tree was achieved by running 1000 bootstrapping replications. Bootstrap values (%) are indicated by numbers at the nodes. The scale for branch length is shown below the tree.

In order to determine the evolutionary status of *Sp*FMRFamide, we constructed a phylogenetic tree by the maximum likelihood method (MEGA 6.0) (Figure 3) that contains 45 types of known FLPs or FaRPs amino acid sequences from vertebrates, annelids, arthropods, and mollusks. This phylogenetic analysis indicated that the tree consists of two apparent branches, one was made up of vertebrate FLP sequences, and the other one was made up of invertebrate FLP sequences. The branch containing invertebrate FLP sequences included the FaRP subfamily and the LFRFamide subfamily. *Sp*FMRFamide and FMRFamides from *S. japonica* and *S. officinalis* belonged to the same sub-branch, suggesting they are close relatives [35].

### 2.3. Quantitative Expression Analysis of SpFMRFamide

**Figure 4:**
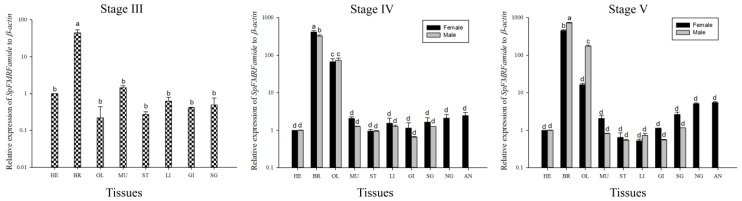
Expression analysis of *SpFMRFamide* from various tissues in three different stages (III, IV, and V). In each case, the expression level of the heart was regarded as the reference value. Vertical bars represented the mean ± standard deviation (SD) (*n* = 3). The analyses were conducted in eight/ten different tissues including the heart (HE), brain (BR), optic lobe (OL), muscle (MU), stomach (ST), liver (LI), gill (GI), sexual gland (SG, including testis and ovary), nidamental gland (NG), and accessory nidamental gland (AN). *β-actin* served as a reference gene and a, b, c, and d on the bars represent statistically significant differences among the tissues in the Least Significant Difference (LSD) multiple comparison test of the Statistical Product and Service Solutions (SPSS) (*p* < 0.05).

The relative expression of *SpFMRFamide* mRNA in the tissues was measured by qRT-PCR. We divided the development periods of the cuttlefish *S. pharaonis* into six stages based on the published articles [36,37]. The differential expression of *SpFMRFamide* transcripts at three different developmental stages for both male and female cuttlefish is shown in Figure 4. In each case, the expression level of the heart was regarded as the reference value. The data is normalized, and the expression level of *SpFMRFamide* in the heart was set to = 1. Tissue expression analysis confirmed that *SpFMRFamide* was widely distributed among tissues and predominantly expressed in the brain at the three development stages. In stage III, trace amounts of expressed *SpFMRFamide* were found in the optic lobe; however, in stage IV, the FMRFamide was significantly expressed in both the female and the male optic lobe of *S. pharaonis* (*p* < 0.05). In stage III, the relative expression in the brain was approximately more than 45 times, and the expression was significantly higher in the brain than in other tissues (*p* < 0.05). In stage IV, the expression in the brain of females and males was approximately 413 times and 323 times, respectively, and the expression in the optic lobes of females and males was 66 times and 73 times, respectively. The mRNA expression of *SpFMRFamide* in the brain was higher in the female cuttlefishes than in male cuttlefishes. The expression was significantly higher in the brain and optic lobes than those in other tissues (*p* < 0.05). In stage V, the expression in the brain of females and males was approximately 448 times and 723 times, and the expression in the optic lobes of females and males was 16 times and 173 times. The mRNA expression of *SpFMRFamide* in the brain was higher in the male cuttlefishes than in female cuttlefishes, which is opposite to the results of expression in stage IV. And the mRNA expression of *SpFMRFamide* in the optic lobe was significantly higher in the male cuttlefishes than in female cuttlefishes (*p* < 0.05).

### 2.4. Effects of SpFMRFamide and SpFLRFamide on the Total Protein Secretory Activity of CHO-K1 Cells

**Figure 5:**
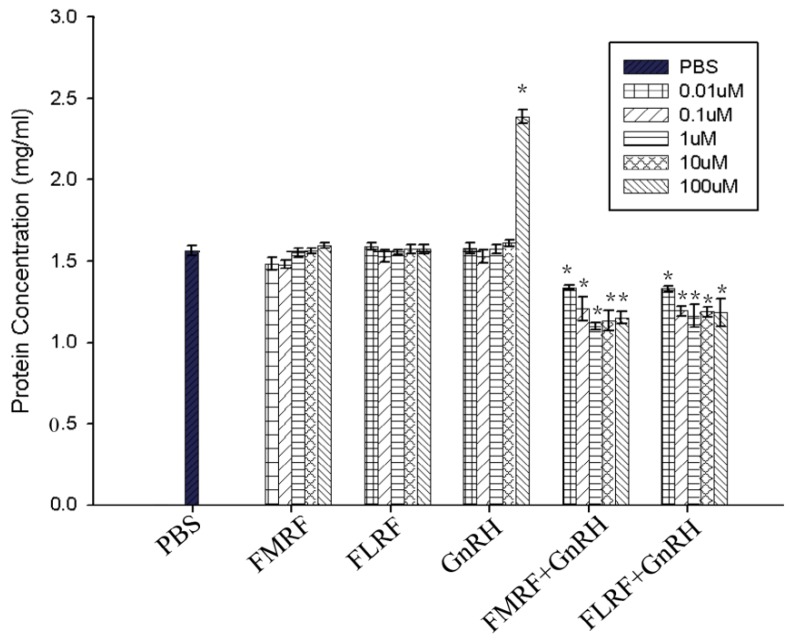
The effect of *Sp*FMRFamide, *Sp*FLRFamide, or *Sp*GnRH and the associated effect of *Sp*FMRFamide+*Sp*GnRH or *Sp*FLRFamide+*Sp*GnRH on protein secretion in CHO-K1. Statistical significance of results was analyzed by one-way ANOVA followed by Tukey’s post-tests. Significant differences (*p* < 0.05) are noted by ‘*’ over each concentration group compared to the control (phosphate buffer saline treated, PBS group).

After neuroactive substances, namely *Sp*FMRFamide, *Sp*FLRFamide, or *Sp*GnRH, were added to incubate with CHO-K1 cells, different effects were observed depending on the total protein secretion of the CHO-K1 cells; in addition, the combined effect of *Sp*FMRFamide or *Sp*FLRFamide with *Sp*GnRH was also tested. The results showed that the 0.01 µM, 0.1 µM, 1 µM, 10.0 µM, and 100 µM final concentrations of both *Sp*FMRFamide and *Sp*FLRFamide had no significant inhibitory effect on the secretion of total protein in CHO-K1, respectively (*p* < 0.05). In contrast, when the dodecapeptide *Sp*GnRH was used as a stimulating agent (final concentration of 100 µM), the total protein secretion of CHO-K1 cells increased significantly (*p* < 0.05). Finally, the combined effects of *Sp*FMRFamide+*Sp*GnRH and *Sp*FLRFamide+*Sp*GnRH showed a marked decrease in the level of total proteins released in the supernate and the treatment of 1.0 μM (final concentration of both peptides) showed the most obvious inhibition effect, possibly due to the presence of FMRFamide or FLRFamide (Figure 5).

## 3. Discussion

A growing body of research has uncovered that FMRFamide may play roles in the regulation, feeding, secretion, migration, invasion, and reproductive regulation and processes [30,38]. Moreover, FMRFamide plays a regulatory role in nerve cells. For example, the FMRFamide neuropeptide plays a vital role in *S. officinalis* pigment regulation. In addition, FMRFamide is involved in several physiological functions in other species [30,39], including insects, other mollusks, amphibians, birds, and mammals. Some studies have suggested that the *Sp*FMRFamide neuropeptide might be similar in function to that of other cephalopods regarding life activities [40].

The results of the phylogenetic tree analysis indicated that the precursors of FMRFamide of *S. pharaonis*, *S. japonica*, and *S. officinalis* belong to the same clade, with the closest relationship as compared to other taxa, and the amino acid identity of the three species is up to 97%. Therefore, we speculated that utilizing the same living environment, exhibiting similar feeding habits, frequent distribution and exchange in common areas of the sea, and the long-term effects of environmental conditions might contribute to similar evolutionary trajectories. In addition, the precursors of FMRFamide of *S. pharaonis, D. opalescens, D. pealeii,* and *I. notoides* are in the same clade based on the high amino acid sequence similarity, indicating that the precursors of FMRFamide in cephalopods may have originated from a common ancestor. Secondly, *S. pharaonis* clustered with other mollusks, including *A. californica* and *L. stagnalis*. However, we know little about the FMRFamide polypeptides in cephalopods, thus, the evolution of the FMRFamide neuropeptide requires additional analysis.

The data analysis of qRT-PCR suggested that the *SpFMRFamide* transcripts were detected in both male and female *S. pharaonis* brains during the three developmental stages. Our results confirmed the conclusion that *SpFMRFamide* is expressed early in life, but whether they play roles during mollusk development and play different mechanisms in male and female cuttlefish needs confirmation with the following experiments.

In *O. vulgaris*, FMRFamide regulates sexual maturity by inhibiting gland activity [27]. In *S. pharaonis*, the action mechanisms of FMRFamide are not clear, but we could presume that *Sp*FMRFamide might control spawning activity directly by regulating the transmission of oocytes in the oviduct. For example, FMRFamide and FLRFamide stimulate tubal contraction, while FIRFamide and ALSGDAFLRFamide weaken tonic contraction, intensity, and frequency [41]; the interactions and relations between neuropeptides and other hormones also deserve attention. Based on the results of this study, we concluded that *Sp*FMRFamide might be involved in the regulation of reproductive activity and other activities. By observing FMRFamide and gonadotropin-releasing hormone (GnRH) immunoreactivity that targeted the optic lobes of *O. vulgaris*, Di Cosmo and Di Cristo [42] found that there may be some relation between the two neuropeptides, indicating that the neuropeptides of cephalopods might exhibit cross-actions associated with physiological activities. In the paper, CHO-K1 was used for the in vitro incubation of neuropeptides. The experimental results showed that a certain dose range of *Sp*FMRFamide and SpFLRFamide had no inhibitory effect on the total amount of protein secretion in CHO-K1, respectively (*p* < 0.05). Interestingly, when each of tetrapeptides with *Sp*GnRH was tested on CHO-K1 cells, the treated samples showed a marked decrease in the amount of proteins released in the medium, possibly due to the presence of FMRFamide and FLRFamide. Inhibition of FMRFamide and FLRFamide does not seem to work unless they cooperate with other neuroactive substances [43,44]. According to this phenomenon, we intend to design the next series of experiments to explore the function of FMRFamide and FLRFamide in the signal pathway [42,45].

## 4. Materials and Methods

### 4.1. Sample Collection

Healthy cuttlefish (*S. pharaonis*), representing three developmental stages (stage III, stage IV, and stage V that were divided based on the growth time and gonad appearance) [36,37] were collected from an aquaculture base (Shacheng, Fuding, Fujian Province, China). Cuttlefish representing the three stages were cultured in filtered seawater at approximately 25 °C with an artificial photoperiod of 14 h light and 10 h dark per day, and specimens were fed shrimp twice daily. All the in vivo tests were carried out at the School of Marine Science and Technology of Zhejiang Ocean University (Zhoushan, China), which obtained permission for performing the research protocols and all animal experiments conducted during the present study from the ethics committee of Zhejiang Ocean University. All experimental procedures were conducted under the oversight and approval of the Academy of Experimental Animal Center of Zhejiang Ocean University and in strict accordance with the NIH Guide for the care and use of laboratory animals (NIH, 2002).

### 4.2. RNA Extraction and cDNA Full-Length Amplification

The RNA extraction and cDNA full-length amplification were based on previously described methods [46]. The cuttlefish were anesthetized with ice for 1 min before dissection, and then tissues of muscle, heart, gill, stomach, liver, testis, nidamental gland, accessory nidamental gland, ovary, brain, and optic lobe were removed separately and saved with RNAlater (Sangon Biotech, Shanghai, Co., Ltd., China) at −80 °C. Trizol lysis buffer (Takara Bio Inc., Otsu, Kyoto, Japan) was used to obtain total RNA from different tissues, and the RNA samples were reverse transcribed into first-strand cDNA with reverse transcriptase (Takara Bio Inc.). The first-strand cDNA of mature cuttlefish was used as the template.

According to the known conserved sequences of *S. japonica* (KJ933411), *S. officinalis* (Y11246.1), *L. stagnalis* (U03137.1), *D. pealeii* (FJ205479.1), *D. opalescens* (AF303160.1), and *I. notoides* (FJ896403.1), FMRF-F/FMRF-R degenerate primers (Table 1) were designed. The PCR amplification reaction was conducted with Takara Taq DNA polymerase (Takara Bio Inc.). The target band was purified from PCR products, then transferred to the p Ucm-T Vector (Bio Basic, Inc., Markham, ON, Canada), and sent to sequence determination. Once we got the core sequence of *SpFMRFamide*, gene-specific primers (FMRF-5′ outer and FMRF-5′ inner, FMRF-3′ outer and FMRF-3′ inner; Table 1) were designed to amplify the two ends. RACE reactions were conducted with the guidance of instructions of First Choice RLM RACE Kit (Ambion, Austin, TX, USA), and then the RACE PCR products were transferred to the p Ucm-T Vector (Bio Basic, Inc., Markham, ON, Canada) in order to sequence determination.

### 4.3. Bioinformatics Analysis of SpFMRFamide

The bioinformatics analysis of *SpFMRFamide* cDNA sequence was analyzed with previously described methods [46] including the following aspects: amino acid coding region, the potential signal sequences, and the MW prediction, theoretical isoelectric point calculation (*pI*), multiple sequence alignments based on amino acid of the precursor proteins and nucleotide sequences of the genes, and phylogenetic tree analysis based on the sequence of the precursor proteins by the maximum likelihood method (MEGA 6.0). The software or websites used were Lasergene software (DNASTAR, Inc., Madison, Wisconsin, USA), Scratch Protein Predictor, and Predict Protein websites, SignalP v3.0, Blastx and Blastn, ClustalW2, and ExPASy ProtParam online tool.

### 4.4. Quantitative Real-Time PCR of SpFMRFamide in Different Tissues

qRT-PCR was performed to study the expression levels of *SpFMRFamide* in three development stages and various tissues of the cuttlefish on the basis of specific primers. The work was done and based on previously described methods [46] with the primers RT-FMRF-F, RT-FMRF-R, and 7500 real-time PCR System (Applied Biosystems, UK) and SYBR premix Ex TaqTM II kit (Perfect Real Time) (Takara Bio, Inc.). The *β-actin* gene (RT-actin-F and RT-actin-R; Table 1) was set as the internal reference. Three individuals were taken for each tissue, and each tissue was repeated three times. PCR specificity was analyzed on the basis of dissociation curve.

The mRNA expression level detected in the heart tissue was used as the reference value. The threshold and Ct (threshold cycle) values acquired via qRT-PCR were used to analyze *SpFMRFamide* mRNA levels according to the 2^−ΔΔCt^ method. All data were normalized and are presented as mean ± SD (*n* = 3). The data were subjected to the LSD multiple comparison test of SPSS. Differences were considered significant at *p* < 0.05, and different letters on the bars represent statistically significant differences among the tissues.

### 4.5. Protein Secretion Activity of SpFMRFamide, SpFLRFamide, and SpGnRH in CHO-K1 Cells

The Chinese hamster ovary cell strain K1 (CHO-K1) was bought from Shanghai Institute of Cellular Biology of the Chinese Academy of Sciences. The peptides of *Sp*FMRFamide, *Sp*FLRFamide, and *Sp*GnRH used in the experiment (Table 2) were manually synthesized by Shanghai bioengineering company with solid-phase peptide synthesis method. The purity of the peptide was checked by analytical reverse-phase high-performance liquid chromatography (RP-HPLC) using a Beckman HPLC system (System Gold) coupled to a UV detector. The predicted mass of peptide used in this study was checked by an electrospray ionization mass spectrometer. The amino acid sequence of GnRH mature dodecapeptide is from *S. pharaonis* (GenBank accession number: MT211953).

The cells were cultured in cell culture flasks until the logarithmic growth phase, then the medium was discarded, and cells were washed twice with PBS, and digested by trypsin. After the cells were adjusted to a cell suspension of 1 × 10^4^/well, inoculated 180 μL/well to a 96-well plate, and incubated in a 5% CO_2_ incubator at 37 °C for 24 h 20 μL peptide solution (final concentrations of 0.01 μΜ, 0.1 μM, 1 μM, 10 μM, and 100 μM, respectively, for both peptides) was mixed in the treatment groups, while PBS (excluding serum and double resistance) was as a blank group. The absorbance at 562 nm of the supernatant was measured after 24 h by an enzyme labeling apparatus, and the standard curve was made by BCA means to determine the concentration of secreted protein.

## 5. Conclusions

In conclusion, our study represents the first report describing the full-length sequence *SpFMRFamide*. Moreover, the results of qRT-PCR and in vivo assay were used to assess the possible function of *Sp*FMRFamide in *S. pharaonis*. The results of this study support cephalopod phylogeny. With the investigation of *S. pharaonis*, as with further species of cuttlefish, it contributes answers to open questions about the role of FMRF peptides in biological processes. Furthermore, the study could be of theoretical interest and practically useful for the renewal of marine resources. Further investigations are necessary to confirm the significance of FMRFamide in reproductive regulation and other activities, and the phylogenetic relations among FMRFamide precursors of cephalopod species.

## Figures and Tables

**Table 1:** Primers used in this study.

Primer Name	Sequence (5′-3′)	Positions
FMRF-F	GCTGAAGATGAAYTGGAAGARGAC	1019–1042
FMRF-R	TCATCRTCTTCACCKCCGCG	1235–1254
FMRF-5′outer	GATGTCTCTAGCTTGCTTCC	1336–1355
FMRF-5′inner	GATAGTGCTGAACAGAAAAAC	764–784
FMRF-3′outer	GAAACCCTGAGGACCAAGAAGCTGACAA	1188–1215
FMRF-3′ inner	GGCGGTGAAGACGATGACGTGAACT	1238–1262
RT-actin-F	TGAGAGGGAGATTGTGCGTG	815–834
RT-actin-R	GAACATAGATTCTGGAGCACGG	968–989
RT-FMRF-F	CCTGAGGACCAAGAAGCTGA	1193–1212
RT-FMRF-R	GCCAAAGAAGGAAGCAAGCT	1345–1364
FMRF-probeF	CCCAAGCGTGATGCGTTGTTGGAGT	331–349
FMRF-probeR	CCGGAACGTTTGCTTCTCAGTCCATC	515–534

**Table 2:** Peptides used in the present study.

Peptide	Sequence
*Sp*FMRFamide	FMRF-NH_2_
*Sp*FLRFamide	FLRF-NH_2_
*Sp*GnRH	pENYHFSNGWHPG-NH2
